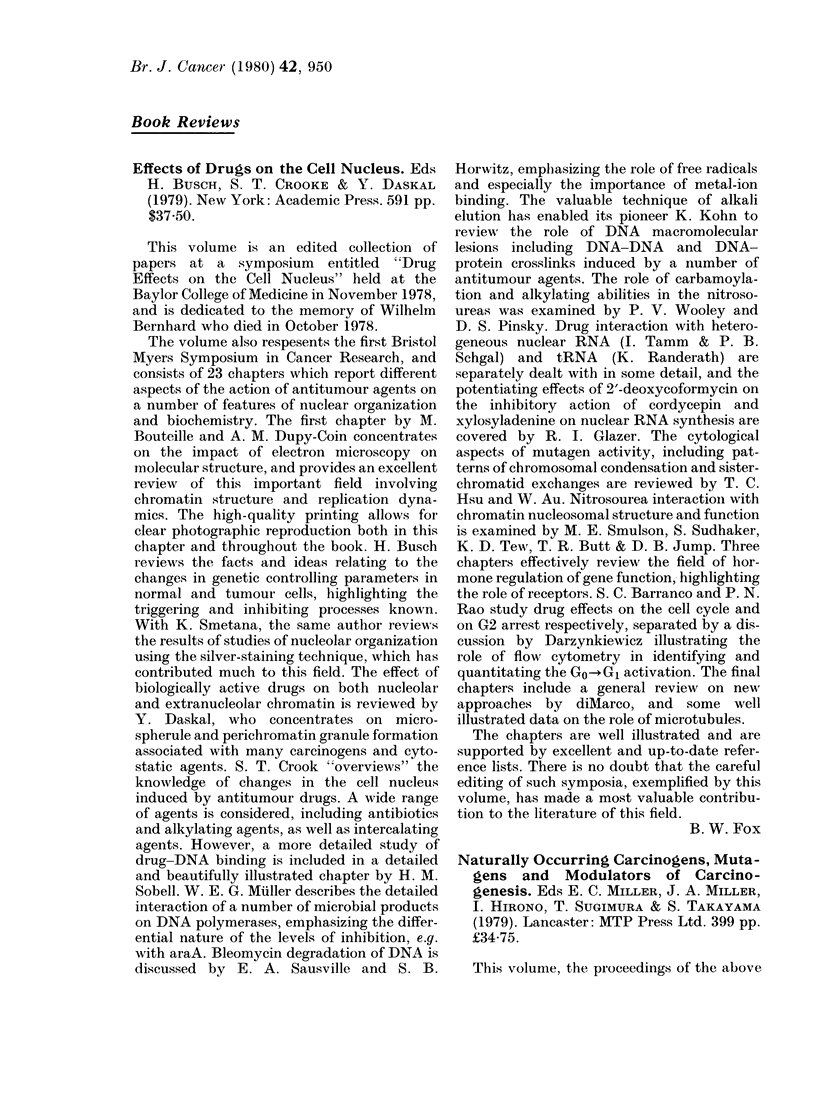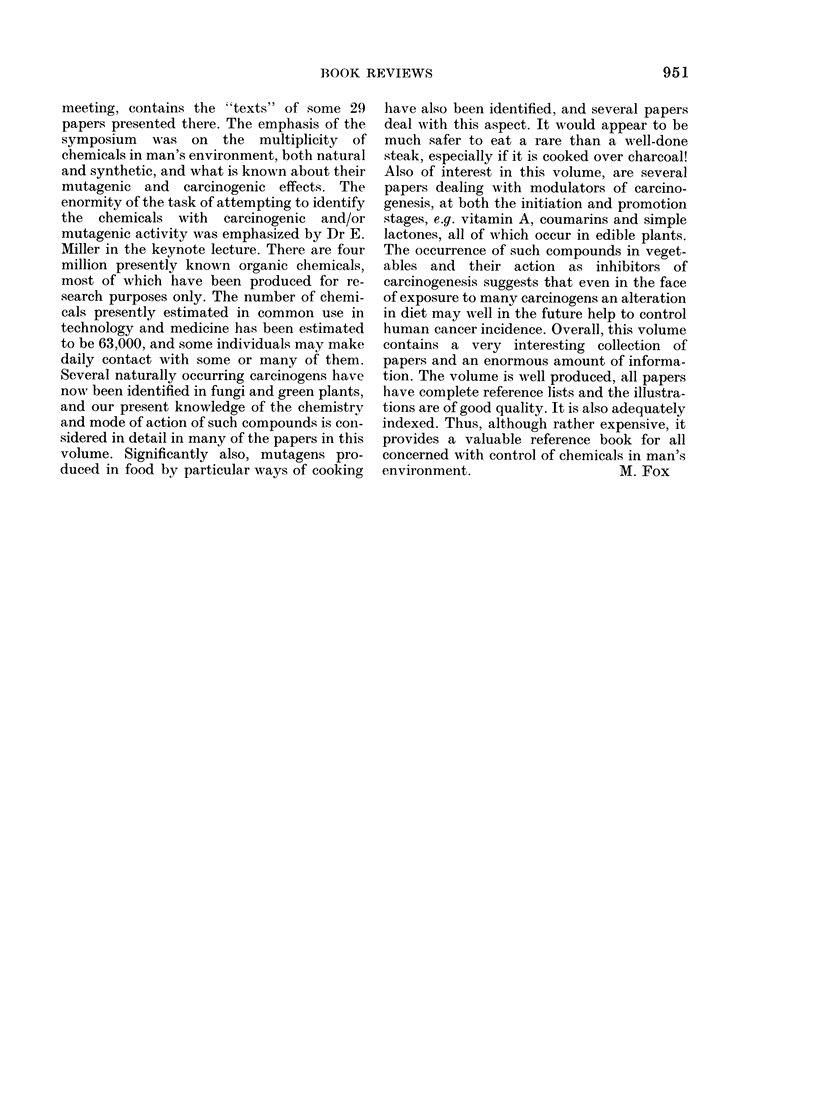# Naturally Occurring Carcinogens, Mutagens and Modulators of Carcinogenesis

**Published:** 1980-12

**Authors:** M. Fox


**Naturally Occurring Carcinogens, Mutagens and Modulators of Carcinogenesis.** Eds E. C. Miller, J. A. Miller, I. Hirono, T. Sugimura & S. Takayama (1979). Lancaster: MTP Press Ltd. 399 pp. £34·75.

This volume, the proceedings of the above meeting, contains the "texts" of some 29 papers presented there. The emphasis of the symposium was on the multiplicity of chemicals in man's environment, both natural and synthetic, and what is known about their mutagenic and carcinogenic effects. The enormity of the task of attempting to identify the chemicals with carcinogenic and/or mutagenic activity was emphasized by Dr E. Miller in the keynote lecture. There are four million presently known organic chemicals, most of which have been produced for research purposes only. The number of chemicals presently estimated in common use in technology and medicine has been estimated to be 63,000, and some individuals may make daily contact with some or many of them. Several naturally occurring carcinogens have now been identified in fungi and green plants, and our present knowledge of the chemistry and mode of action of such compounds is considered in detail in many of the papers in this volume. Significantly also, mutagens produced in food by particular ways of cooking have also been identified, and several papers deal with this aspect. It would appear to be much safer to eat a rare than a well-done steak, especially if it is cooked over charcoal! Also of interest in this volume, are several papers dealing with modulators of carcinogenesis, at both the initiation and promotion stages, *e.g.* vitamin A, coumarins and simple lactones, all of which occur in edible plants. The occurrence of such compounds in vegetables and their action as inhibitors of carcinogenesis suggests that even in the face of exposure to many carcinogens an alteration in diet may well in the future help to control human cancer incidence. Overall, this volume contains a very interesting collection of papers and an enormous amount of information. The volume is well produced, all papers have complete reference lists and the illustrations are of good quality. It is also adequately indexed. Thus, although rather expensive, it provides a valuable reference book for all concerned with control of chemicals in man's environment.